# Supplementary material for: The effect of coronary stent policies on the risk of percutaneous coronary intervention among acute coronary syndrome patients in Shanghai: Real-world evidence
Source: PLoS One. 2024 Apr 1;19(4):e0301448. doi: 10.1371/journal.pone.0301448 (PMC10984406; doi:10.1371/journal.pone.0301448)
Supplement: S1 Table — (DOCX) [file pone.0301448.s002.docx]

S1 Table. Cox model for restenosis or stent thrombosis 1 year after PCI

|  | Influencing factors ^a^ | Parameter estimate | Standard error | χ^2^ | p value | Hazard ratio |
| --- | --- | --- | --- | --- | --- | --- |
| Policy implementation | | 0.2841 | 0.7185 | 0.16 | 0.6926 | 1.329 |
| Age (years) | |  |  |  |  |  |
|  | 60- | -1.9501 | 1.1396 | 2.93 | 0.0870 | 0.142 |
|  | 70- | -1.1633 | 0.9334 | 1.55 | 0.2127 | 0.312 |
| Male | | -0.9748 | 0.7723 | 1.59 | 0.2069 | 0.377 |
| No insurance | | -0.2852 | 1.1305 | 0.06 | 0.8008 | 0.752 |
| Medical history | |  |  |  |  |  |
| Diabetes | | -0.4255 | 0.8871 | 0.23 | 0.6315 | 0.653 |
| Hypertension | | 0.0530 | 0.8206 | 0.00 | 0.9485 | 1.054 |
| Hyperlipidemia | | -0.4997 | 1.0387 | 0.23 | 0.6305 | 0.607 |
| Stroke | | -16.2588 | 16272.00 | 0.00 | 0.9992 | 0.000 |
| Chronic kidney disease | | 0.3135 | 1.1892 | 0.07 | 0.7920 | 1.368 |
| COPD | | 1.8429 | 1.1732 | 2.47 | 0.1162 | 6.315 |
| MI | | -15.9775 | 2579.00 | 0.00 | 0.9951 | 0.000 |
| CABG | | 2.4908 | 1.2906 | 3.73 | 0.0536 | 12.071 |
| Tertiary hospital | | -1.3376 | 0.7725 | 3.00 | 0.0834 | 0.262 |
| NYHA or Killip functional classification (reference: I) | |  |  |  |  |  |
|  | IV | -16.7081 | 8445.00 | 0.00 | 0.9984 | 0.000 |
|  | II or III | -0.2609 | 0.7866 | 0.11 | 0.7402 | 0.770 |
| Number of coronary stents implanted (reference: 1) | |  |  |  |  |  |
|  | 2 | 0.7510 | 0.7324 | 1.05 | 0.3051 | 2.119 |
|  | 3 | -16.0425 | 5900.00 | 0.00 | 0.9978 | 0.000 |
|  | 4 or more | -16.7144 | 19362.00 | 0.00 | 0.9993 | 0.000 |
| Outpatient medicines used within 1 year after PCI (reference: Other medicine) | |  |  |  |  |  |
|  | Aspirin | -0.1087 | 0.8456 | 0.02 | 0.8977 | 0.897 |
|  | Clopidogrel | 1.2433 | 0.7842 | 2.51 | 0.1129 | 3.467 |
|  | Metoprolol | -1.3024 | 0.7578 | 2.95 | 0.0857 | 0.272 |
|  | Ticagrelor | 1.5607 | 0.8081 | 3.73 | 0.0535 | 4.762 |
|  | Atorvastatin | -0.8999 | 0.8173 | 1.21 | 0.2708 | 0.407 |
|  | Ezetimibe | -0.5992 | 1.1290 | 0.28 | 0.5956 | 0.549 |
|  | Sacubitril/valsartan | -0.3589 | 1.1237 | 0.10 | 0.7495 | 0.698 |
|  | Trimetazidine dihydrochloride | -15.6466 | 3641.00 | 0.00 | 0.9966 | 0.000 |
|  | Pravastatin sodium | 0.3189 | 1.1484 | 0.08 | 0.7812 | 1.376 |
|  | Nicorandil | -16.6210 | 4825.00 | 0.00 | 0.9973 | 0.000 |
|  | Isosorbide dinitrate | -0.2084 | 1.1090 | 0.04 | 0.8509 | 0.812 |
|  | Rosuvastatin | 0.2725 | 0.8254 | 0.11 | 0.7413 | 1.313 |
| Testing global null hypothesis | |  |  |  |  |  |
|  | Likelihood ratio |  |  | 26.78 | 0.6833 |  |
|  | χ^2^ _Wald_ |  |  | 21.93 | 0.8853 |  |

^a^ All the independent variables in the models were 1-0 variables (1 for “yes”, 0 for “no”); n=6375.
