# Supplementary material for: The effect of coronary stent policies on the risk of percutaneous coronary intervention among acute coronary syndrome patients in Shanghai: Real-world evidence
Source: PLoS One. 2024 Apr 1;19(4):e0301448. doi: 10.1371/journal.pone.0301448 (PMC10984406; doi:10.1371/journal.pone.0301448)
Supplement: S2 Table — (DOCX) [file pone.0301448.s003.docx]

S2 Table. Cox model for MI 1 year after PCI

|  | Influencing factors ^a^ | Parameter estimate | Standard error | χ^2^ | p value | Hazard ratio |
| --- | --- | --- | --- | --- | --- | --- |
| Policy implementation | | -0.2837 | 0.2020 | 1.97 | 0.1603 | 0.753 |
| Age (years) | |  |  |  |  |  |
|  | 60- | -0.1635 | 0.2685 | 0.37 | 0.5427 | 0.849 |
|  | 70- | 0.2111 | 0.2671 | 0.62 | 0.4293 | 1.235 |
| Male | | 0.5297 | 0.2663 | 3.96 | 0.0467 | 1.698 |
| No insurance | | -0.1725 | 0.3519 | 0.24 | 0.6240 | 0.842 |
| Medical history | |  |  |  |  |  |
| Diabetes | | 0.0475 | 0.2205 | 0.05 | 0.8294 | 1.049 |
| Hypertension | | 0.2432 | 0.2377 | 1.05 | 0.3061 | 1.275 |
| Hyperlipidemia | | -0.2369 | 0.2392 | 0.98 | 0.3219 | 0.789 |
| Stroke | | -0.4080 | 1.0149 | 0.16 | 0.6876 | 0.665 |
| Chronic kidney disease | | -0.0816 | 0.3254 | 0.06 | 0.8019 | 0.922 |
| COPD | | 0.1087 | 0.4712 | 0.05 | 0.8175 | 1.115 |
| MI | | 0.2062 | 0.3561 | 0.34 | 0.5625 | 1.229 |
| CABG | | -0.1883 | 0.6123 | 0.09 | 0.7585 | 0.828 |
| Tertiary hospital | | -0.1926 | 0.2847 | 0.46 | 0.4986 | 0.825 |
| NYHA or Killip functional classification (reference: I) | |  |  |  |  |  |
|  | IV | 1.0902 | 0.3610 | 9.12 | 0.0025 | 2.975 |
|  | II or III | 0.0001 | 0.2143 | 0.00 | 0.9998 | 1.000 |
| Number of coronary stents implanted (reference: 1) | |  |  |  |  |  |
|  | 2 | -0.1457 | 0.2427 | 0.36 | 0.5483 | 0.864 |
|  | 3 | 0.1007 | 0.4636 | 0.05 | 0.8280 | 1.106 |
|  | 4 or more | 0.8552 | 0.7238 | 1.40 | 0.2374 | 2.352 |
| Outpatient medicines used within 1 year after PCI (reference: Other medicine) | |  |  |  |  |  |
|  | Aspirin | -0.1610 | 0.2277 | 0.50 | 0.4795 | 0.851 |
|  | Clopidogrel | 0.1058 | 0.2402 | 0.19 | 0.6596 | 1.112 |
|  | Metoprolol | -0.4978 | 0.2027 | 6.03 | 0.0140 | 0.608 |
|  | Ticagrelor | 0.0429 | 0.2427 | 0.03 | 0.8596 | 1.044 |
|  | Atorvastatin | -0.0673 | 0.2550 | 0.07 | 0.7920 | 0.935 |
|  | Ezetimibe | -0.6701 | 0.3152 | 4.52 | 0.0335 | 0.512 |
|  | Sacubitril/valsartan | 1.2543 | 0.2114 | 35.22 | <.0001 | 3.505 |
|  | Trimetazidine dihydrochloride | -0.4587 | 0.4653 | 0.97 | 0.3243 | 0.632 |
|  | Pravastatin sodium | -1.5037 | 0.7224 | 4.33 | 0.0374 | 0.222 |
|  | Nicorandil | 0.5596 | 0.3146 | 3.16 | 0.0753 | 1.750 |
|  | Isosorbide dinitrate | 0.3436 | 0.2500 | 1.89 | 0.1693 | 1.410 |
|  | Rosuvastatin | -0.1865 | 0.2911 | 0.41 | 0.5218 | 0.830 |
| Testing global null hypothesis | |  |  |  |  |  |
|  | Likelihood ratio |  |  | 78.33 | <.0001 |  |
|  | χ^2^ _Wald_ |  |  | 80.58 | <.0001 |  |

^a^ All the independent variables in the models were 1-0 variables (1 for “yes”, 0 for “no”); n=6375.
