# Supplementary material for: The effect of coronary stent policies on the risk of percutaneous coronary intervention among acute coronary syndrome patients in Shanghai: Real-world evidence
Source: PLoS One. 2024 Apr 1;19(4):e0301448. doi: 10.1371/journal.pone.0301448 (PMC10984406; doi:10.1371/journal.pone.0301448)
Supplement: S3 Table — (DOCX) [file pone.0301448.s004.docx]

S3 Table. Cox model for death 1 year after PCI

|  | Influencing factors ^a^ | Parameter estimate | Standard error | χ^2^ | p value | Hazard ratio |
| --- | --- | --- | --- | --- | --- | --- |
| Policy implementation | | -0.3426 | 0.4734 | 0.52 | 0.4693 | 0.710 |
| Age (years) | |  |  |  |  |  |
|  | 60- | 16.4253 | 1634.00 | 0.00 | 0.9920 | 1.36E+07 |
|  | 70- | 16.4722 | 1634.00 | 0.00 | 0.9920 | 1.42E+07 |
| Male | | 1.5750 | 0.7651 | 4.24 | 0.0395 | 4.831 |
| No insurance | | 1.0512 | 0.8358 | 1.58 | 0.2085 | 2.861 |
| Medical history | |  |  |  |  |  |
| Diabetes | | 0.6346 | 0.4832 | 1.72 | 0.1891 | 1.886 |
| Hypertension | | 1.6035 | 0.8083 | 3.94 | 0.0473 | 4.970 |
| Hyperlipidemia | | -1.1921 | 0.5489 | 4.72 | 0.0299 | 0.304 |
| Stroke | | 0.8221 | 1.0802 | 0.58 | 0.4466 | 2.275 |
| Chronic kidney disease | | -1.4021 | 1.0524 | 1.78 | 0.1828 | 0.246 |
| COPD | | 1.0125 | 0.8018 | 1.59 | 0.2067 | 2.752 |
| MI | | 0.4107 | 0.5945 | 0.48 | 0.4896 | 1.508 |
| CABG | | 0.5808 | 0.8074 | 0.52 | 0.4719 | 1.787 |
| Tertiary hospital | | -0.8809 | 0.5856 | 2.26 | 0.1325 | 0.414 |
| NYHA or Killip functional classification (reference: I) | |  |  |  |  |  |
|  | IV | 1.7962 | 0.8449 | 4.52 | 0.0335 | 6.027 |
|  | II or III | 0.8033 | 0.5122 | 2.46 | 0.1168 | 2.233 |
| Number of coronary stents implanted (reference: 1) | |  |  |  |  |  |
|  | 2 | -0.8817 | 0.6384 | 1.91 | 0.1672 | 0.414 |
|  | 3 | -15.8058 | 2477.00 | 0.00 | 0.9949 | 0.000 |
|  | 4 or more | -17.2477 | 10196.00 | 0.00 | 0.9987 | 0.000 |
| Outpatient medicines used within 1 year after PCI (reference: Other medicine) | |  |  |  |  |  |
|  | Aspirin | -1.8971 | 0.6194 | 9.38 | 0.0022 | 0.150 |
|  | Clopidogrel | -0.3805 | 0.5782 | 0.43 | 0.5105 | 0.684 |
|  | Metoprolol | -1.1889 | 0.5652 | 4.43 | 0.0354 | 0.305 |
|  | Ticagrelor | -0.9348 | 0.6219 | 2.26 | 0.1328 | 0.393 |
|  | Atorvastatin | 0.0031 | 0.5297 | 0.00 | 0.9953 | 1.003 |
|  | Ezetimibe | -16.4359 | 2001.00 | 0.00 | 0.9934 | 0.000 |
|  | Sacubitril/valsartan | 0.8291 | 0.5491 | 2.28 | 0.1311 | 2.291 |
|  | Trimetazidine dihydrochloride | -16.4617 | 3426.00 | 0.00 | 0.9962 | 0.000 |
|  | Pravastatin sodium | 0.9692 | 0.7863 | 1.52 | 0.2178 | 2.636 |
|  | Nicorandil | -0.1282 | 1.0542 | 0.01 | 0.9032 | 0.880 |
|  | Isosorbide dinitrate | -0.3257 | 0.7692 | 0.18 | 0.6720 | 0.722 |
|  | Rosuvastatin | 0.9521 | 0.6411 | 2.21 | 0.1375 | 2.591 |
| Testing global null hypothesis | |  |  |  |  |  |
|  | Likelihood ratio |  |  | 88.21 | <.0001 |  |
|  | χ^2^ _Wald_ |  |  | 49.02 | <.0001 |  |

^a^ All the independent variables in the models were 1-0 variables (1 for “yes”, 0 for “no”); n=6375.
